# Mental Health Literacy Levels of Medical Staff in China: An Assessment Based on a Meta-Analysis

**DOI:** 10.3389/fpsyt.2021.683832

**Published:** 2021-11-05

**Authors:** Shengyu Guo, Jie Xiong, Feiyue Liu, Yanlin Su

**Affiliations:** ^1^Department of Economics and Management, Changsha University, Changsha, China; ^2^Department of Mathematics and Computer Science, Changsha University, Changsha, China; ^3^Department of Gynaecology and Obstetrics, The Affiliated Changsha Central Hospital, University of South China, Changsha, China

**Keywords:** awareness rate, medical staff, mental health, knowledge, meta-analysis

## Abstract

**Background:** The awareness rate of mental health knowledge among medical staff is an important evaluation index to assess the service capacity of a country or region, and this indicator in China has not been quantitatively evaluated.

**Study Design:** This study systematically combined pertinent quantitative study data from previous related studies to conclude the awareness rate of mental health knowledge among Chinese medical staff.

**Methods:** Related studies from five electronic databases were searched, and a meta-analysis was conducted to obtain the combined result. The primary outcome of the present study was the awareness rate of medical staff or the sample size and the number of those who can answer the relevant questions correctly. We also performed a hierarchical analysis according to the sample group's region and specialty. The awareness rate of medical staff and corresponding 95% confidence intervals (CIs) were calculated. The heterogeneity was assessed with the *I*^2^ test, and Egger's test was used to evaluate publication bias.

**Results:** A total of 15 articles with 11,526 medical staff were included in the present study; the overall awareness rate of mental health knowledge among Chinese medical staff was as low as 81%. The awareness rate of mental health knowledge among medical workers in developed regions is higher than that in developing regions. The awareness rate of mental health among medical staff in the department of psychiatry, non-psychiatry, and community medical staff was 88, 68, and 82%, respectively.

**Conclusion:** The overall awareness rate among medical staff in this country is unsatisfactory, and the awareness rate in developed regions is higher than medical staff in developing regions. Psychiatric hospital staff has a higher awareness rate than community medical staff, and non-psychiatric hospital staff has the lowest awareness rate.

## Introduction

At present, COVID-19 continues to ravage the world, with 200 million people having been infected and more than 3 million people dying from the disease. However, the damage that the epidemic has brought to us does not end there; the outbreak has also led to a significant rise in mental disorders (1). Mental disorders have become an important public health problem all around the world in recent years (2). In the United States, the direct cost spent in treating different types of mental illness is as high as $148 billion per year, accounting for 2.5% of its gross national product (GNP) (3). In China, mental illness is the main source of disease burden, accounting for 30% of the total disease burden (4). Studies indicated that if proper interventions cannot be implemented as soon as possible, the burden of mental illness will increase greatly in China (5). The COVID-19 public health emergency has exacerbated this adverse situation; it is estimated that the epidemic has led to a significant increase in the incidence of common mental disorders, such as depression, anxiety, and emergency syndrome (6). As a developing country, China is faced with the serious shortage and uneven distribution of mental health resources (7). Although the government of this country has drawn up a series of plans to deal with the challenge of mental disorders, many factors, such as effective policies, adequate mental health service system, and high quality of medical staff influence the goals. The awareness rate of mental health knowledge is an important evaluation indicator that reflects the quality of mental health services in a country (8). A previous study indicated that the awareness rate of mental health knowledge among medical staff is also an evaluation index to assess the mental health service capacity of a country or region (9). A number of studies have concluded that the poor awareness rate of medical staff (about mental health knowledge) is significantly associated with a high rate of misdiagnosis among mental patients (10, 11). In countries like China, where mental health resources are scarce, medical staff may be the main professional resource that patients can rely on (12). With high-quality medical staff, the service of mental health can be effectively promoted. Especially at a time when the COVID-19 epidemic has not been effectively controlled, high-quality healthcare groups are an important force for safeguarding people's physical and mental health. In order to improve the capacity of mental health services and meet the people's mental health needs, the Chinese government issued an important document in 2015, called the Chinese national mental health work plan (2015–2020), which formulated a series of action plans, including improving the coordination mechanism of comprehensive mental health management, reforming the mental health service system, and strengthening the management of mental health work. According to the plan, by the end of 2020, the awareness rate of mental health knowledge among urban and rural people should be up to 70 and 50%, respectively, and the awareness rate of students at school should be up to 80% at least (13). The prerequisite for achieving this goal is to make sure that medical staff meet the requirement, as they not only play an important role in the diagnosis and treatment of mental illness patients, but also a vital role in the health education of common groups (11). Thus, it is important to evaluate the awareness rate of mental health knowledge among Chinese medical staff, so we can understand the real gap between the goals and reality and provide the basis for future mental health promotion plans. Although some researchers have carried out relevant studies, some limitations of previous studies should be addressed: (1) the awareness rate of medical staff varies greatly among different studies, so a merged rate is in need; (2) all previous studies were limited in local regions or cities, without a study of nationwide scale; and (3) the sample size of relative studies was small and could not have sufficient statistical effect (14).

To address these limitations, we subjected the included studies to a statistical procedure of meta-analysis to integrate the results of several independent studies, obtain a more objective appraisal of the evidence, and provide a more precise estimate of a research effect (15). Thus, a meta-analysis of related studies was conducted in this study, so we can provide valuable information for improving mental health services in China.

## Methods

### Search Strategy

Five electronic databases (Web of Science, PubMed, WanFang, CNKI, and WeiPu) were searched for related studies from the inception of the databases up until July 2019. Only studies published in English or Chinese were considered. Search terms in PubMed was “awareness rate” (MeSH), “knowledge of mental health” (MeSH), and “medical staff” (MeSH), and China. In CNKI, the advanced search mode was used, with “mental health knowledge” and “medical group” as the main subject terms.

### Study Selection

Studies were included in the analyses if the following criteria were satisfied: (i) The subjects were medical staff, which include community medical staff defined as “community health workers who worked in a community health service center,” psychiatric staff defined as “a doctor or nurse practicing psychiatry,” and non-psychiatric staff defined as “a non-psychiatric practitioner in a general hospital.” (ii) Research content was mental health awareness rate. (iii) Study subjects were from China, and according to the level of economic development, this study classifies the following regions as developed regions: Beijing, Shanghai, Guangdong province, and Zhejiang province; other regions were considered as developing regions. (iv) Only cross-sectional studies were included. (v) The questionnaire used was based on the mental health knowledge content recommended by the national health administration (13). The questionnaire consists of three parts; in the first part, there were 16 items, which mostly consist of mental health knowledge, including information about mental health, such as Mental Health Day, Suicide Prevention Day, World Sleep Day, and mental health knowledge acquisition. The second part of the questionnaire is about attitudes toward mental illness and its sufferers, with 11 items set up as questions to reflect attitudes toward mental illness and patients, such as “Would you like to make friends with someone with a mental illness or believe that someone with a mental illness can return to the community?” The third part of the questionnaire consists of five case studies, each consisting of 11 items (presented as questions). This part further reflects the knowledge of the respondents about mental diseases, including the etiology and management methods of common mental disorders such as neurasthenia, depression, mania, and schizophrenia.

### Data Extraction and Bias Assess

Two authors independently extracted relevant data from the included studies. These data consisted of name of first author, year of publication, study design, research location, sample size, awareness rate or the number of people who can answer the relevant questions correctly, the specialty of medical staff, size of hospital, psychiatric hospital or not, professional psychiatrist or not, and community medical staff or not. Medical staff in this study was defined as professional health technicians, which refers to professional technical personnel engaged in medical services, including doctors and nurses.

The first main outcome of this study is the awareness rate of medical staff, which was defined as the number of medical staff who can answer the questions correctly divided by the sample size. The second outcome is the sample source, whether it was a psychiatric hospital or not, whether the medical staff are professional psychiatrists or not, or whether they are community medical staff or not. We assessed the risk of bias according to the guidelines of the Cochrane reviews (16). Two of our authors conducted the evaluation independently on such information: representativeness of sample, the consistence of survey tools, and information integrity. Included studies were classified as “low risk of bias,” “unclear risk of bias,” or “high risk of bias,” with respect to the above information.

### Statistical Analysis

Five transformations (including the original rate, logarithmic transformation of the original rate, perform logit conversion to the original rate, inverse sine transform of the original rate, and Freeman–Tukey double inverse sine conversion of the original rate) were used to evaluate whether the distribution of the main outcome (awareness rate of medical staff) conforms to the normal distribution (17). Furthermore, the transformed rate that is closest to the normal distribution was selected to perform rate merging. The awareness rate of the medical staff and the corresponding 95% confidence intervals (CIs) were calculated. The heterogeneity was assessed with the *I*^2^-test, with an *I*^2^ > 50% indicating the existence of heterogeneity (if *I*^2^ > 50%, the random model was adopted; if *I*^2^ < 50%, the fixed model was adopted) (18). In addition, the publication biases of articles involved were evaluated by funnel plot and confirmed using Egger's test. All statistical analyses were conducted using R version 3.4.4 (R Project for Statistical Computing, Vienna, Austria). Statistical tests were considered significant when *p* < 0.05.

## Results

### Study Selection

**Figure 1:**
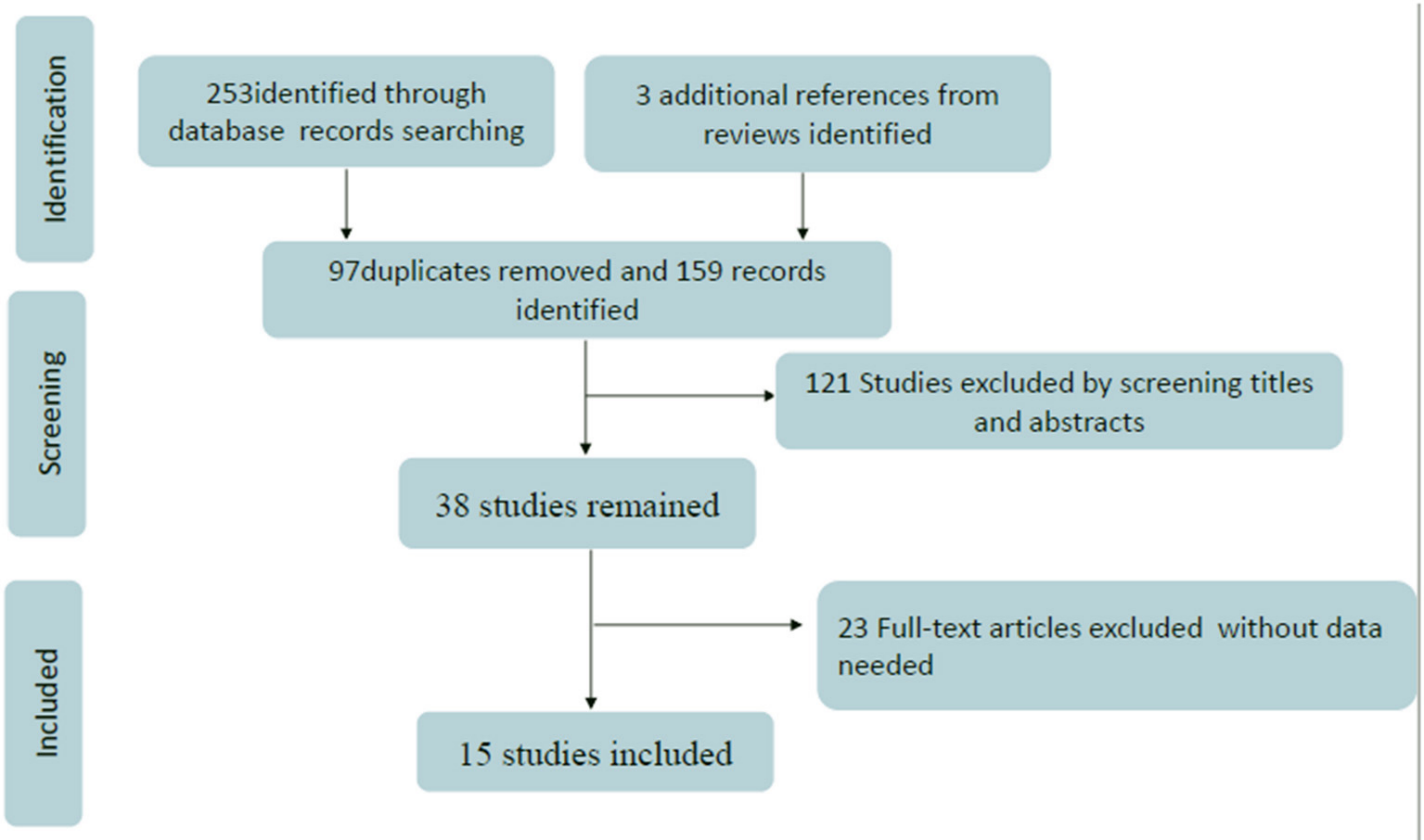
Flowchart presenting the steps of the literature search and selection.

We searched the relative database according to the search strategy, and obtained 256 potentially eligible trials. Then 97 duplicates were removed, and another 121 studies were excluded after screening the titles and abstracts. Among the remaining 38 full-text studies, 23 studies were ruled out due to the absence of a valuable outcome. Finally, a total of 15 articles with 11,526 medical staff were included in the present study to evaluate the awareness rate of mental health knowledge among Chinese medical staff (19–33). Due to the limited information in the included literature, this study only studied the items reflecting the knowledge of mental health, and the attitude about the mental illness and patients were not involved in this study. The flowchart is schematically shown in Figure 1.

### Study Characteristics

The general characteristics of the included studies are shown in Table 1. A total of 15 studies were included in this study, including 11,526 medical staff that came from various levels of medical institution in China. All the studies contained the information of the investigation about the related knowledge of medical staff, so the awareness rate was regarded as the main observation target of this study. Among the identified articles, there were 10 studies that researched the awareness rate of community medical staff, including 7,838 community health workers (about 68% of the total sample). The other six studies were about medical staff in hospitals, including 3,334 non-psychiatrists (about 28.93% of the total sample) and 354 psychiatrists (about 3.07% of the total sample). The quality of the included research literature was moderate, and the detailed results of the bias assessment is shown in Table 2.

**Table 1:** The basic information and data of all included studies in the meta-analysis.

**Study/Author**	**Year**	**Responders**	**Sample size**	**Sample type**	**Area**	**Regional**
Zhang (17)	2005	1,368	2,345	Non-psychiatric hospital staff	Shanghai	Developed region
Cheng (18)	2016	292	328	Community medical staff	Beijing	Developed region
Hu (19)	2016	253	432	Non-psychiatric hospital	Yunnan	Developing region
Wang (20)	2018	792	938	Community medical staff	Hebei	Developing region
Wu (21)	2015	496	620	Community medical staff	Guangzhou	Developed region
Cui (22)	2015	865	1028	Community medical staff	Hebei	Developing region
Zhu (23)	2018	137	147	Psychiatric hospitall stall	Zhejiang	Developed region
Tan (24)	2017	80	108	Psychiatric hospitall stall	Xizhang	Developing region
Liu (25)	2010	655	771	Community medical staff	Hunan	Developing region
Lan (26)	2014	91	99	Psychiatric hospitall stall	Shichuan	Developing region
Lan (26)	2014	458	557	Non-psychiatric hospitall stall	Shichuan	Developing region
Huang (27)	2018	183	318	Community medical staff	Ningxia	Developing region
Zheng (28)	2011	109	138	Community medical staff	Shanghai	Developed region
Deng (29)	2014	1886	2167	Community medical staff	Shanxi	Developing region
Yang (30)	2017	765	900	Community medical staff	Hubei	Developing region
Zhou (31)	2017	497	630	Community medical staff	Hubei	Developing region

**Table 2:** Risk of bias assessment.

**Study**	**Sample representativeness**	**Consistence of survey tools**	**Information integrity**	**Total score**
Zhang (17)	L	L	L	3
Cheng (18)	L	L	L	3
Hu (19)	U	L	L	2
Wang (20)	U	L	L	2
Wu (21)	H	L	L	2
Cui (22)	L	L	L	3
Zhu (23)	L	L	L	3
Tan (24)	L	L	L	3
Liu (25)	L	L	L	3
Lan (26)	H	L	L	2
Huang (27)	H	L	L	2
Zheng (28)	L	L	L	3
Deng (29)	H	L	L	2
Yang (30)	L	L	L	3
Zhou (31)	U	L	L	2

*L, indicated low risk; H, high risk; U, unclear risk*.

### Overall Awareness Rate of Medical Staff in China

**Figure 2:**
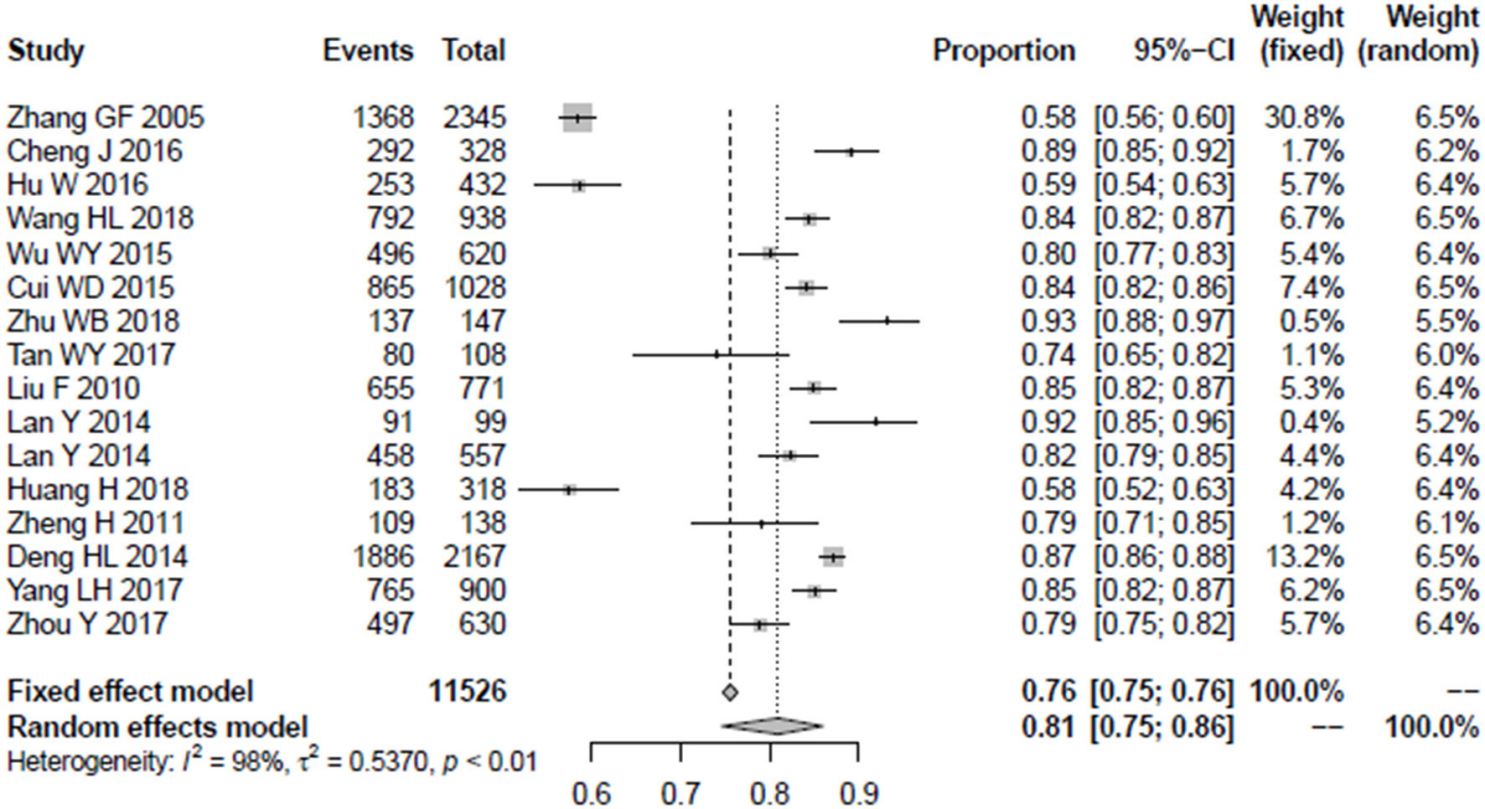
The forest plot of overall awareness rate among medical staff.

The normality test indicated that logit conversion of the original rate was closest to the normal distribution, so this was applied to the original rate before we merged the results. The detailed results of the Shapiro–Wilk normality test is shown in Table 3. The heterogeneity was assessed with the *I*^2^-test, with an *I*^2^ > 50% indicating the existence of heterogeneity, and according to the result of Figure 2, there was a significant heterogeneity in this result, so the random model was selected, and the combined awareness rate of mental health knowledge among Chinese medical staff was 81%, and the 95% confidence interval was 75–86%.

**Table 3:** The detailed result of Shapiro–Wilk normality test.

**Method**	***W*-value**	***P*-value**
*P*(original rate)	0.842n	0.011
Log(*p*)	0.802	0.002
Logit(*p*)	0.932	0.257
Arcsin(*p*)	0.887	0.051
Darcsin(*p*)	0.890	0.055

*P, the original rate; log, logarithmic transformation of the original rate; logit, perform logit conversion to the original rate; Arcsin, inverse sine transform of the original rate; Darcsin, Freeman–Tukey double inverse sine conversion of the original rate*.

### The Awareness Rate of Medical Staff in Different Development Regions

**Figure 3:**
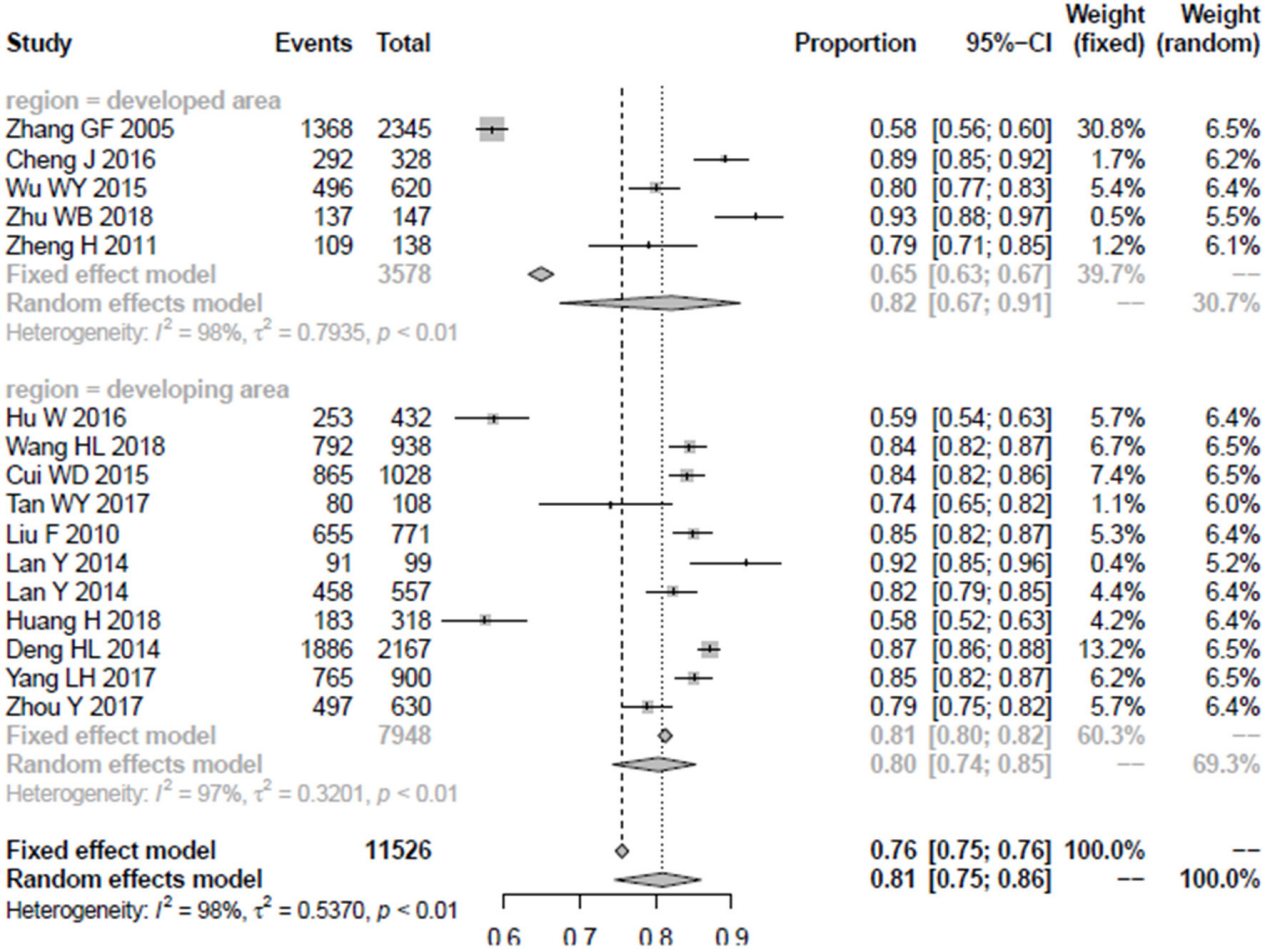
The forest plot of awareness rate among developed and developing regions.

In order to compare the mental health awareness rate of medical staff in regions with different levels of development, this study divides the whole country into developed and developing regions. Beijing, Shanghai, Guangdong province, Zhejiang province, and Jiangsu province were considered as developed regions, while other parts of the country were classified as developing regions. A total of 5 articles covering 3,578 medical staff were conducted in developed regions, while 10 other studies including 7,948 medical staff were conducted in developing regions. For the developed regions, the random model was selected due to the existence of heterogeneity (*I*^2^ = 98%), and the combined rate (and its 95% confidence interval) was 82% (0.67–0.91). The combined rate of medical staff in developing regions was 80% (95% CI = 0.74–0.85). The detailed results are shown in Figure 3.

### The Awareness Rate of Different Medical Professionals

**Figure 4:**
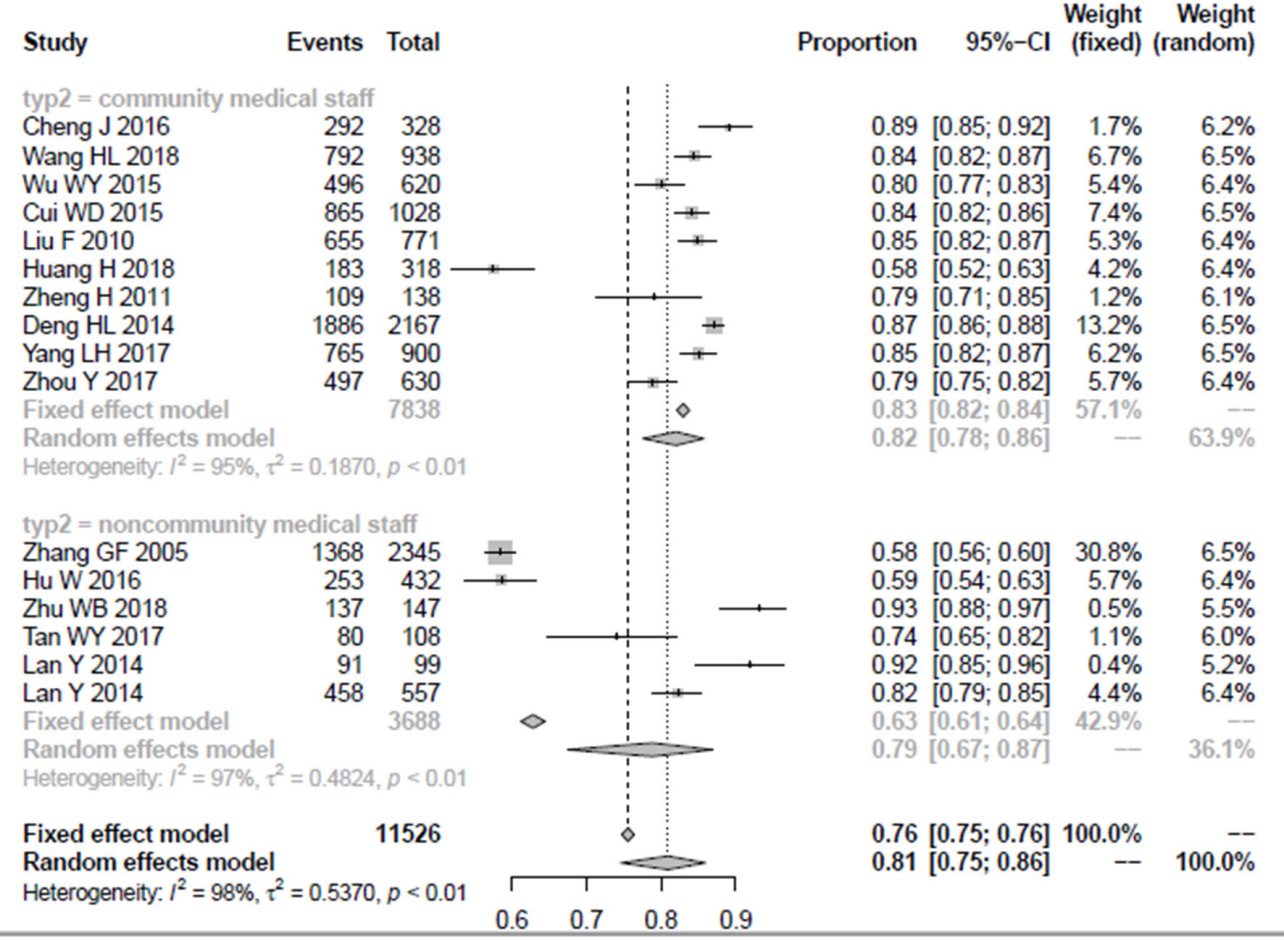
The forest plot of awareness rate among community medical staff and non-community medical staff.

In this study, 10 articles included 7,838 community medical staff as the research sample, and the combined rate was almost the same as the overall awareness rate of medical staff in China, 82 (95% CI = 0.78–0.86). A total of 3,688 non-community medical staff were the research sample in five studies; due to significant heterogeneity, the random model was applied, and the results indicated that the awareness rate was lower than the community medical staff 79 (95% CI = 0.67–0.87). The detailed results are shown in Figure 4.

**Figure 5:**
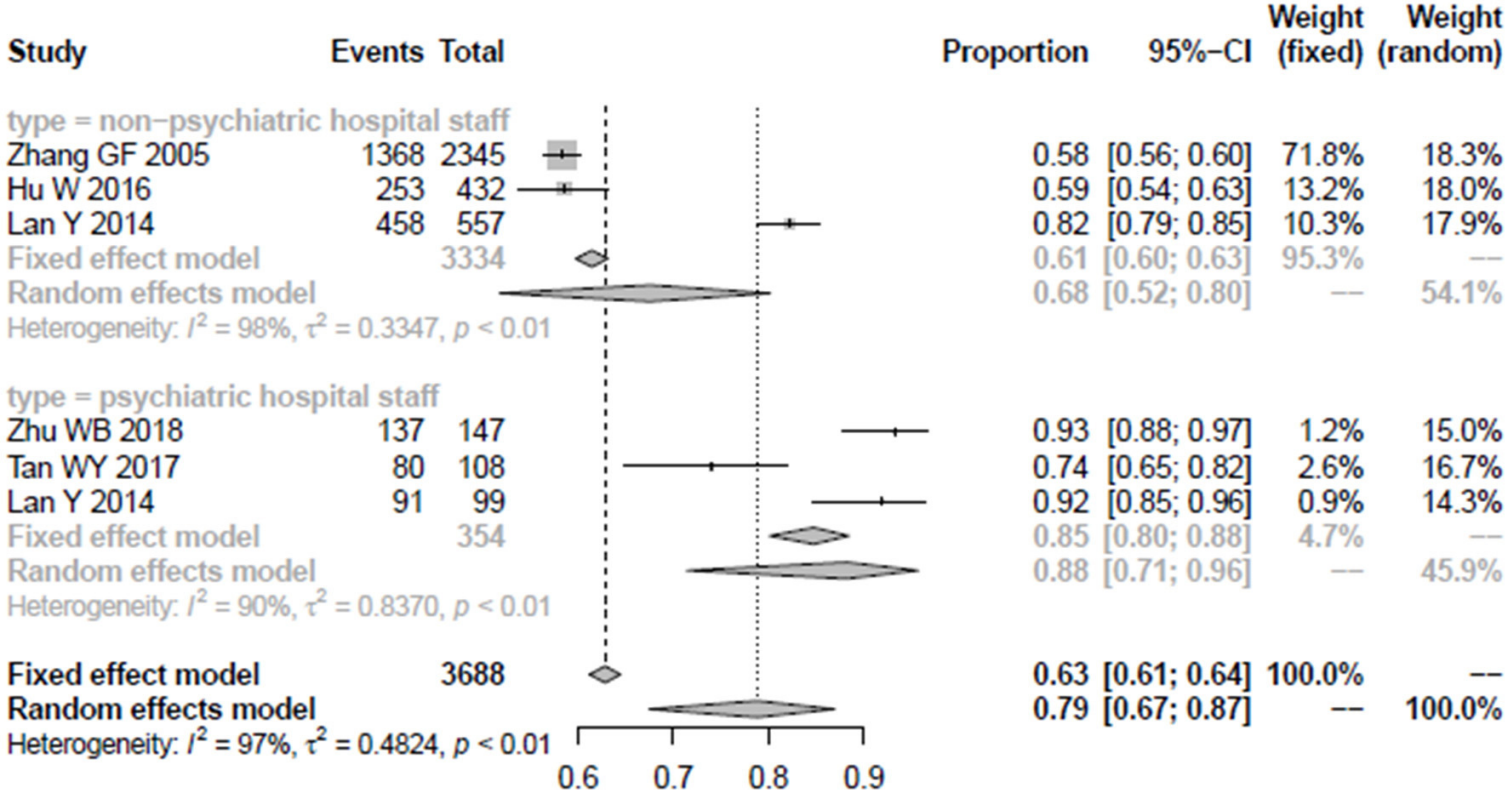
The forest plot of awareness rate among psychiatric staff and non-psychiatric staff.

Among the five articles that take non-community medical staff as the research sample, there were three studies with psychiatric staff as the research sample, while another three articles include 3,334 non-psychiatric staff as the research sample; among the two groups, their awareness rate and 95% confidence interval were 88% (0.71–0.96) and 68% (0.52–0.80), respectively. The detailed results are shown in Figure 5.

### Publication Bias

**Figure 6:**
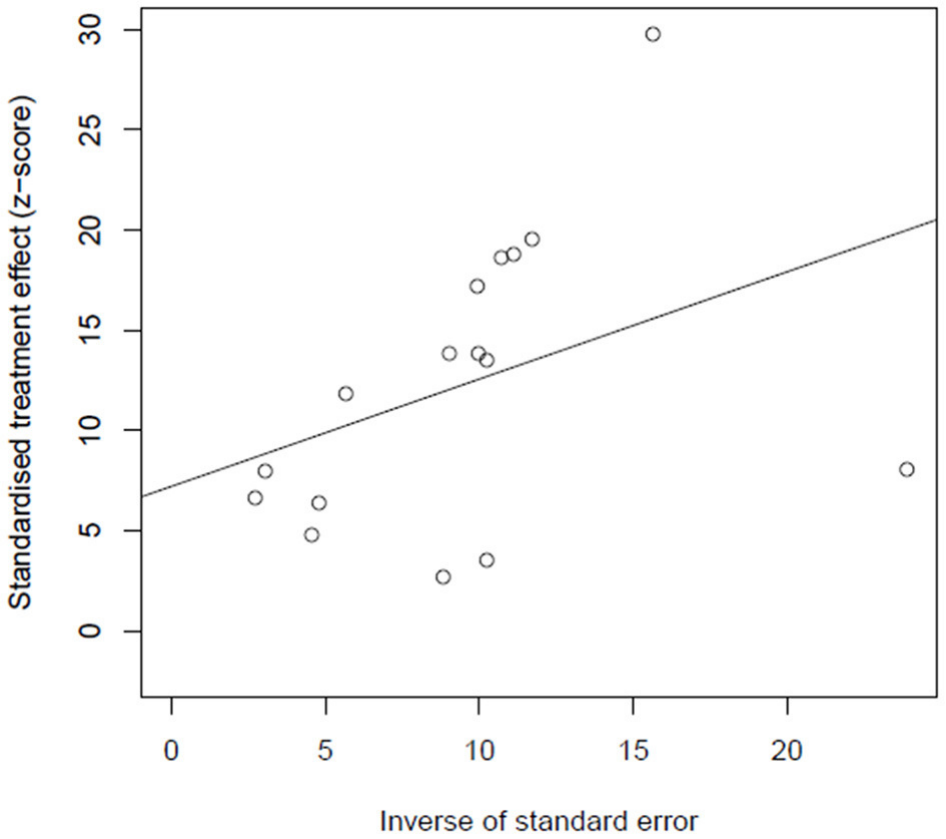
Funnel plot of publication bias.

Egger's', test was used to identify whether publication bias exists in the study; there was no significant publication bias identified (*t* = 1.93, *p* = 0.07, bias = 7.21, se.bias = 3.73, slope = 0.53). Thus, the validity and credibility of this meta-analysis were confirmed. The funnel plot is shown in Figure 6.

## Discussion

The COVID-19 epidemic has caused enormous challenge to mental health services all over the world; the strain on mental health services in many countries has soared (34). During the past decade, the mental health resources in China has increased greatly; in some developed regions, the number of psychiatrists has reached 5.5/100,000 (35), which is almost to the level of some developed countries. Easy-access mental health services have been established all over the country, which can provide relative mental health services to patients. The quality of medical staff undoubtedly was the most important factor that affects the efficiency of service (36). However, the country still faces an urgent need for quality mental health services amid the COVID-19 pandemic; published literatures have concluded that the current mental health service system could not effectively meet the mental health needs of the population (37). Many healthcare workers have been overwhelmed for long periods of time in fighting the epidemic; they also face serious mental health challenges (38). Knowledge of mental health may help them respond quickly to such adverse situations. So, in this study, we conducted a meta-analysis to evaluate the awareness rate of mental health knowledge among Chinese medical staff, which was an important evaluation index to assess the capacity of mental health service. Moreover, due to occupational factors, medical staff have a higher incidence of anxiety and depression than the general population (39); thus, ideal mental health knowledge not only helps to maintain the mental health of this group but also helps them to provide quality medical services for their patients. Previous studies mostly focused on the mental health status of healthcare groups (11, 40), and few studies explored the underlying mechanism from the perspective of mental health knowledge. According to the theory model of knowledge–belief–behavior (41), behavior is directly influenced by belief and indirectly influenced by knowledge, so the awareness rate of mental health knowledge is very important for medical staff.

The combined result indicated that the overall awareness rate among medical staff in this country was lower, only attaining the level of the general population (national mental health work plan (13)). Experiences of developed countries suggested that the training of mental health knowledge to medical staff should be reinforced (42). Medical teams with good mental health knowledge can not only help diagnose and treat mental diseases but also effectively guide people to correctly understand mental diseases, eliminate discrimination and stigma, and promote standard treatment (43). The awareness rate in developed regions is higher than that in developing regions (82% for developed regions, 80% for developing regions, *p* < 0.05), which suggests that mental health service capacity of medical staff needs to be greatly improved in developing regions where resources are insufficient. Some researchers have identified higher rates of mental illness and high suicide rates of mental disorders in remote parts of the country (44), which means more professional mental health services are needed in these areas.

We also conducted a meta-analysis on the awareness rate of medical staff working in hospitals and communities. Interestingly, the result shows that community health workers have a higher awareness rate than those who work in hospitals, though hospitals generally provide more care than community health services in China (82% for community medical staff, 79% for non-community medical staff, *p* < 0.05). This may be due to the fact that in areas like China, where mental health resources are scarce, primary care is the most accessible facility for patients (9), so community medical staff are more likely to have contact with people with mental disorders. Among different medical service teams, the highest awareness rate comes from psychiatric hospital staff, with an awareness rate of 88%; the awareness rate of non-psychiatric medical staff was only 68%, even lower than the awareness rate of the general population (13). The results indicated that mental health knowledge training for medical staff needs to be established, especially for non-psychiatric medical staff. As the backbone of the mental health service system, the mental health knowledge of medical staff determines the quality of mental health services they provide (45), especially in areas with poor mental health resources.

This is the first meta-analysis to conduct a quantitative analysis and evaluation of mental health knowledge awareness rate of medical personnel in different regions and different specialties in China, although the present study can provide valuable information for enhancing the mental health system and may be beneficial in reducing the burden caused by mental disorders in the country. However, some limitations of this study should be noted, which may affect the results. First, though we had conducted a thorough literature search, all included studies were published in Chinese, which may cause publication bias (although our results show no publication bias). Second, though awareness rate was a predefined outcome, assessment tools are not uniform in different studies, which may influence the result. Third, significant variation existed in the number of studies with respect to each kind of medical staff; for example, the research object of 10 articles is community medical staff, while only three studies were conducted on both psychiatric hospital staff and non-psychiatric hospital staff. So, this may result in some bias.

In summary, the overall awareness rate among Chinese medical staff is 81%, and the awareness rate in developed regions is higher than the medical staff in developing regions, indicating more effort should be directed in developing regions to promote balanced development. Psychiatric hospital staff has a higher awareness rate compared to community medical staff, and non-psychiatric hospital staff has the lowest awareness rate, suggesting that more effective mental health training should be given to medical staff, especially for community medical staff and non-psychiatric hospital staff.

## Data Availability Statement

The original contributions presented in the study are included in the article/supplementary material, further inquiries can be directed to the corresponding author/s.

## Author Contributions

All authors listed have made a substantial, direct and intellectual contribution to the work, and approved it for publication.

## Funding

This work was supported by Education fund of Hunan province (No. 18B409), Hunan Educational Planning Project (No. CJ194001), Research Project of Hunan Provincial Health Commission (No. 20201952), Project of Hunan Society of Health Economics (No. 2021B05), and National Planning Office of Philosophy and Social (No. 17BSH059).

## Conflict of Interest

The authors declare that the research was conducted in the absence of any commercial or financial relationships that could be construed as a potential conflict of interest.

## Publisher's Note

All claims expressed in this article are solely those of the authors and do not necessarily represent those of their affiliated organizations, or those of the publisher, the editors and the reviewers. Any product that may be evaluated in this article, or claim that may be made by its manufacturer, is not guaranteed or endorsed by the publisher.

## Abbreviations

Log: logarithmic transformation of the original rate
logit: perform logit conversion to the original rate
Arcsin: inverse sine transform of the original rate
Darcsin: Freeman–Tukey double inverse sine conversion of the original rate.
